# Supplementary material for: Analysis of the Effect of Mixed Fermentation on the Quality of Distilled Jujube Liquor by Gas Chromatography-Ion Mobility Spectrometry and Flavor Sensory Description
Source: Foods. 2023 Feb 13;12(4):795. doi: 10.3390/foods12040795 (PMC9957257; doi:10.3390/foods12040795)
Supplement: Supplementary file 1 [file foods-12-00795-s001.zip › foods-2173551-supplementary.pdf]

Table S1. The flavor compounds presented in the jujube liquor by GC–IMS.

Count	Compound	CAS	Formula	RI	Rt[sec]	Dt[RIPrel]
	Esters					
1	Ethyl octanoate	64-19-7	$C_{10}H_{20}O_2$	1450.20	1140.72	1.48
2	Ethyl heptanoate	106-30-9	$C_9H_{18}O_2$	1341.40	900.88	1.38
3	Ethyl hexanoate	123-66-0	$C_8H_{16}O_2$	1224.50	703.90	1.79
4	Isoamyl acetate-M	123-92-2	$C_7H_{14}O_2$	1141.60	556.72	1.41
5	Isoamyl acetate-D	123-92-2	$C_7H_{14}O_2$	1141.60	556.72	1.75
6	Ethyl 3-methylbutanoate	108-64-5	$C_7H_{14}O_2$	1080.90	457.79	1.27
7	Ethyl butanoate	105-54-5	$C_6H_{12}O_2$	1053.90	422.51	1.56
8	Isobutyl acetate-M	110-19-0	$C_6H_{12}O_2$	1031.30	395.23	1.34
9	Isobutyl acetate-D	110-19-0	$C_6H_{12}O_2$	1030.50	394.20	1.62
10	Propyl acetate	109-60-4	$C_5H_{10}O_2$	996.70	356.63	1.28
11	Ethyl isobutyrate-M	97-62-1	$C_6H_{12}O_2$	984.60	346.85	1.32
12	Ethyl isobutyrate-D	97-62-1	$C_6H_{12}O_2$	985.90	347.88	1.56
13	Ethyl propanoate-M	105-37-3	$C_5H_{10}O_2$	977.90	341.706	1.28
14	Ethyl propanoate-D	105-37-3	$C_5H_{10}O_2$	977.30	341.192	1.46
15	Ethyl acetate	141-78-6	$C_4H_8O_2$	903.40	288.87	1.34
16	Ethyl formate-M	109-94-4	$C_3H_6O_2$	848.90	255.52	1.06
17	Ethyl formate-D	109-94-4	$C_3H_6O_2$	851.00	256.711	1.22
18	Ethyl lactate	97-64-3	$C_5H_{10}O_3$	1362.50	943.08	1.54
19	Butyl acetate	123-86-4	$C_6H_{12}O_2$	1109.20	500.04	1.61
	Alcohols					
20	1-Hexanol	111-27-3	$C_6H_{14}O$	1359.20	936.42	1.32
21	3-Methyl-1-butanol-M	123-51-3	$C_5H_{12}O$	1225.80	705.89	1.24
22	3-Methyl-1-butanol-D	123-51-3	$C_5H_{12}O$	1226.60	707.04	1.49
23	1-Butanol-M	71-36-3	$C_4H_{10}O$	1163.00	597.66	1.26
24	1-Butanol-D	71-36-3	$C_4H_{10}O$	1163.50	598.64	1.38
25	(E)-2-Pentenal	1576-87-0	C_5H_8O	1152.40	577.01	1.38
26	2-Methyl-1-propanol-M	78-83-1	$C_4H_{10}O$	1109.70	500.87	1.26
27	2-Methyl-1-propanol-D	78-83-1	$C_4H_{10}O$	1110.60	502.40	1.37
28	1-Propanol	71-23-8	C_3H_8O	1055.60	424.72	1.25
29	Ethanol	64-17-5	C_2H_6O	948.60	319.84	1.14
30	Methanol	67-56-1	CH_4O	892.20	281.73	0.98
31	2-Propanol	67-63-0	C_3H_8O	936.30	311.11	1.26
	Aldehydes					
32	Nonanal	124-19-6	$C_9H_{18}O$	1408.20	1041.53	1.48
33	3-Methylbutanal	590-86-3	$C_5H_{10}O$	932.90	308.72	1.40
34	2-Methylpropanal	78-84-2	C_4H_8O	837.00	248.77	1.28
35	Propanal-M	123-38-6	C_3H_6O	822.60	240.83	1.05

36	Propanal-D	123-38-6	C_3H_6O	823.40	241.23	1.15
37	Acetaldehyde	75-07-0	C_2H_4O	768.20	213.04	0.98
38	Butanal	123-72-8	C_4H_8O	893.60	282.58	1.13
	Ketones					
39	3-Hydroxy-2-butanone	513-86-0	$C_4H_8O_2$	1290.40	806.82	1.31
40	2-Heptanone	110-43-0	$C_7H_{14}O$	1246.60	736.86	1.64
41	2,3-Pentanedione	600-14-6	$C_5H_8O_2$	1065.80	437.80	1.20
42	2-Pentanone	107-87-9	$C_5H_{10}O$	1002.00	362.29	1.38
43	Acetone	67-64-1	C_3H_6O	842.00	251.55	1.11
44	2-Butanone	78-93-3	C_4H_8O	918.40	298.80	1.28
45	Cyclopentanone	120-92-3	C_5H_8O	1147.50	567.85	1.33
	Alkenes					
46	Limonene	138-86-3	$C_{10}H_{16}$	1200.30	699.56	1.22
47	Terpinolene	586-62-9	$C_{10}H_{16}$	1299.20	822.05	1.20
	Furans					
48	Furfural	98-01-1	$C_5H_4O_2$	1493-80	1253.96	1.09
	Pyridine					
49	2,6-Dimethylpyridine	108-48-5	C_7H_9N	1246.50	736.83	1.45
	Acids					
50	Acetic acid	64-19-7	$C_2H_4O_2$	1503.70	1281.26	1.14

Note: reserved index (RI), retention time (Rt), drift time (Dt), monomer (M), dimer (D)
